# Cardiac arrest as first presentation of arrhythmogenic left ventricular cardiomyopathy due to Filamin C mutation: a case report

**DOI:** 10.1093/ehjcr/ytab422

**Published:** 2021-11-22

**Authors:** Navneet Kandhari, Shafik Khoury, Elijah R Behr, Chris Miles

**Affiliations:** 1 St Georges University Hospital NHS Foundation Trust, Blackshaw Road, Tooting, London SW17 0QT, UK; 2 Cardiovascular Clinical Academic Group, Molecular and Clinical Sciences Institute, St George’s University of London, Cranmer Terrace, Tooting, London SW17 0RE, UK

**Keywords:** Inherited cardiomyopathies, Filamin C, Arrhythmogenic left ventricular cardiomyopathy, Case report

## Abstract

**Background:**

Arrhythmogenic left ventricular cardiomyopathy (ALVC) is a rare form of arrhythmogenic cardiomyopathy characterized by fibrofatty replacement of left ventricular myocardium, malignant arrhythmia, and sudden cardiac death. The definition incorporates several genetic causes, including pathogenic variation in the Filamin C gene (FLNC). Although awareness of ALVC has improved, identification remains challenging and diagnostic criteria continue to evolve.

**Case summary:**

A 50-year-old athletic male was admitted following an out-of-hospital cardiac arrest due to ventricular tachycardia (VT) whilst playing football. Coronary angiography revealed unobstructed epicardial vessels and the diagnosis of ALVC was suggested by cardiovascular magnetic resonance imaging, which demonstrated a mildly dilated and moderately impaired left ventricle with epicardial late gadolinium enhancement in the basal to mid-lateral walls and subendocardial septum. Initial testing with a cardiomyopathy and arrhythmia gene panel was negative but extended testing uncovered a likely pathogenic variant in FLNC. Subsequently, the patient experienced a recurrence of sustained VT necessitating implantable cardioverter-defibrillator (ICD) therapies, ultimately undergoing a combined epicardial and endocardial VT ablation 4 years after presentation. Six months post-ablation, he was asymptomatic and his arrhythmia rendered quiescent.

**Discussion:**

Arrhythmogenic cardiomyopathy should be considered in the evaluation of an initially unexplained cardiac arrest. This case characterizes the clinical features of a patient with FLNC cardiomyopathy and emphasizes the utility of genetic testing using modern gene panels in patients with comparable phenotypes. We also demonstrate that optimal medical therapy with antiarrhythmic drugs, exercise restriction, ICD insertion, and catheter ablation can be useful in the management of ALVC with positive outcomes

*For the podcast associated with this article, please* visit https://academic.oup.com/ehjcr/pages/podcast

Learning points

- Arrhythmogenic left ventricular cardiomyopathy is a distinct form of arrhythmogenic cardiomyopathy (ACM) characterized by left ventricular (LV) structural, electrical, and genetic abnormalities, including systolic dysfunction and arrhythmia, non-ischaemic LV late gadolinium enhancement, left precordial T-wave inversion, low limb lead QRS voltages, and pathogenic variants in ACM-related genes.
- Phenotype-guided genetic testing can enhance diagnostic certainty, facilitate predictive testing of family members, and occasionally inform risk stratification, such as lowering the threshold for primary prevention defibrillator implantation in patients with Filamin C.
- Catheter ablation is a reasonable approach in ACM patients with ventricular arrhythmia who have not responded to optimal antiarrhythmic drug therapy.

## Introduction

Arrhythmogenic cardiomyopathy (ACM) is a rare heritable disorder characterized by ventricular arrhythmia and fibrofatty replacement of ventricular myocardium.^1^^,^^2^ Classically considered a disease of the right ventricle (RV), biventricular and left ventricular ACM phenotypes are increasingly recognized. The term ‘arrhythmogenic left ventricular cardiomyopathy’ (ALVC) has since been adopted as a distinct clinical entity within the spectrum of ACM.

Given its rarity, clinical features and outcomes of patients with ALVC are scarce and identification can prove challenging. However, pathogenic variants affecting Filamin C (FLNC), an essential protein for myocyte integrity and cell signalling, have been increasingly implicated in patients presenting with characteristic hallmarks of ALVC, including LV predominant disease, malignant arrhythmia, and sudden cardiac death.^3^^,^^4^ In this case report, we discuss the challenges of diagnosis and management in a patient who presented with cardiac arrest and later diagnosed with ALVC.

## Timeline 

**Table:** 

Timeline	Clinical events
Premorbid state	Recent symptoms of exertional pre-syncope; playing 5-a-side football and cycling 60–100 miles regularly
Presentation	Out-of-hospital ventricular tachycardia (VT) arrest whilst playing football
	Coronary angiography: unobstructed epicardial vessels
	Cardiac magnetic resonance imaging: mildly dilated left ventricle (LV) (Left ventricular end diastolic volume 214 mL) with moderately impaired LV systolic function (LVEF 41%) and no regional wall motion abnormalities. Non-dilated right ventricle (RV) with preserved systolic function. Low-grade late gadolinium enhancement within the septum and basal lateral subepicardium
	Implantation of secondary prevention defibrillator
	Started *Bisoprolol 5 mg* *o.d.*
	Blood sample sent for Lamin A (LMNA) gene testing and family referred for clinical screening
26 months	LMNA gene studies negative
	Fluorodeoxyglucose (FDG) positron emission cardiac computed tomography: negative for sarcoid and active inflammation
	Implantable cardioverter-defibrillator (ICD) interrogation: brief runs of non-sustained ventricular tachycardia (NSVT) and one appropriate shock for sustained run of fast VT
	Switched to *Nadolol 80 mg* *b.i.d.*
31 months	ICD interrogation: no recorded ventricular arrhythmia
	Genetic testing performed using a panel of 77 cardiomyopathy and arrhythmia-related genes
41 months	Genetic testing unable to identify a cause
	Echo: mildly dilated LV [left ventricular end-diastolic diameter (LVEDD) 59 mm] with mild-moderately impaired LV systolic function (LVEF 45%). Markedly hypokinetic inferolateral and anterolateral walls. Mildly dilated RV with moderately impaired systolic function
	Nadolol not tolerated due to fatigue, changed to *Sotalol 40 mg* *b.i.d.*
	Started *Ramipril 1.25 mg* *o.d.* for left ventricular systolic dysfunction
42 months	Two episodes of NSVT and one of fast VT (250 b.p.m.) treated by antitachycardia pacing
	Increased *Sotalol to 40/80 mg* *b.i.d.*
	Genetic testing to include Filamin C (FLNC)
52 months	Patient tests positive for likely pathogenic FLNC variant
	One episode of fast VT (cycle length 220 ms) within the ventricular fibrillation defibrillator zone whilst climbing a ski slope, shock delivered
	Increased *Sotalol to 80 mg* *b.i.d.*
	Referred for VT ablation
57 months	Combined epicardial and endocardial VT ablation
58 months	Echo: normal LV size and wall thickness (LVEDD 49 mm) with mildly impaired LV systolic function (LVEF 50–54%). Normal RV size with impaired radial function
63 months	No recurrence of arrhythmia at 6-month follow-up post-ablation

## Case presentation

**Figure 1:**
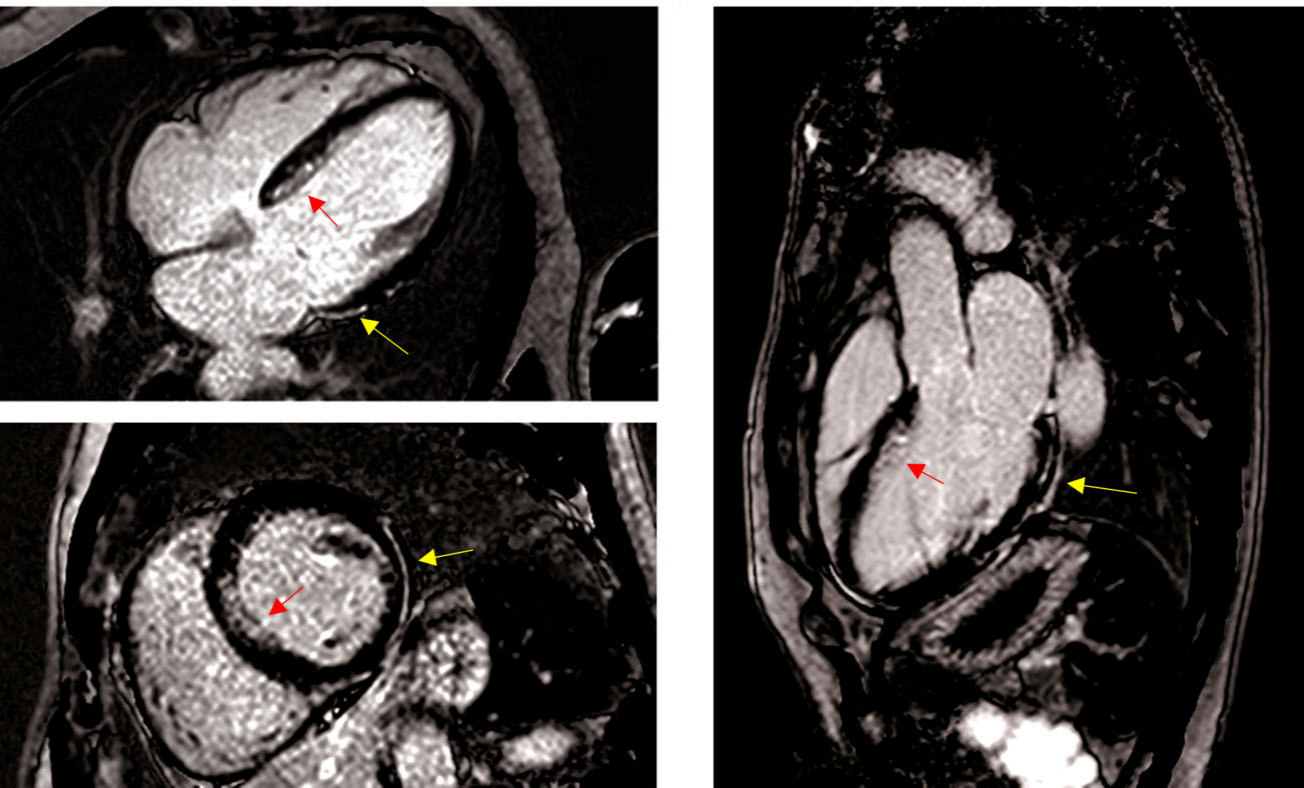
Late gadolinium enhancement cardiovascular magnetic resonance images showing horizontal long axis (four-chamber) (*A*) mid-ventricular short axis (*B*) and left ventricular outflow tract (three-chamber) (*C*) views. The yellow arrows indicate regions of epicardial lateral fibrosis and the red arrows point to septal subendocardial enhancement.

A 50-year-old Caucasian male collapsed unexpectedly whilst playing football. He was brought to hospital following successful resuscitation for an out-of-hospital ventricular tachycardia (VT) arrest. He had no significant past medical or family history of cardiac disease and was a keen recreational athlete, playing 5-a-side football regularly and cycling 60–100 miles per week. In the months leading up to his cardiac arrest, he recalled three brief episodes of exertional pre-syncope whilst playing sports. On admission, coronary angiography showed unobstructed epicardial vessels and echocardiography revealed mild LV impairment with normal heart valves. An FDG PET–computed tomography scan was negative for active myocardial inflammation. However, cardiovascular magnetic resonance imaging (CMRI) demonstrated a mildly dilated and moderately impaired LV [left ventricular ejection fraction (LVEF) 41%] with epicardial late gadolinium enhancement of the lateral wall and subendocardial enhancement of the septum. The RV was non-dilated with no regional wall motion abnormalities (*Figure  1*) (*Videos 1–3*). 

**Figure 2:**
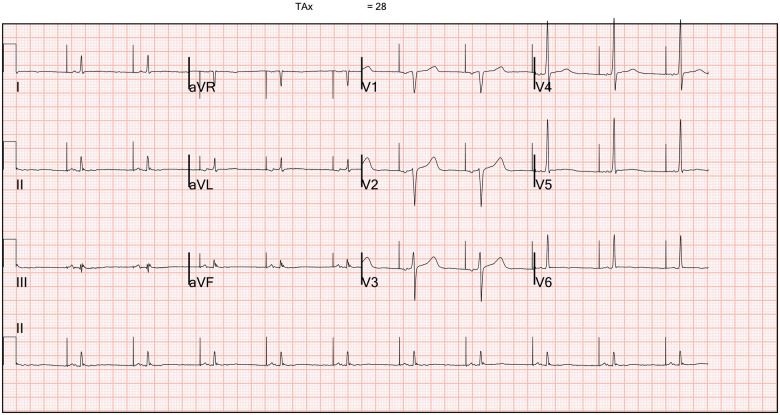
Twelve-lead electrocardiogram post-implantable cardioverter-defibrillator implantation demonstrating an atrial paced rhythm with inferolateral T-wave flattening and low voltage QRS complexes in the limb leads.

A diagnosis of ACM was suspected, and he received an implantable cardiac defibrillator (ICD) for secondary prevention. Post-implantation, his 12-lead electrocardiogram (ECG) showed an atrial paced rhythm with normal ventricular conduction (*Figure  2*). The limb lead complexes were of low voltage and demonstrated fractionated QRS complexes. There were flattened inferolateral T waves and his signal-averaged ECG was negative for late potentials. He was discharged on Bisoprolol 5 mg daily, lifestyle advice to limit his exercise intensity, and referred for genetic testing along with a recommendation for clinical screening of his family members. He was asymptomatic for a significant period, but at 26 months follow-up device interrogation demonstrated three runs of non-sustained ventricular tachycardia (NSVT) and he received one shock for sustained fast VT. He was then trialled on Nadolol therapy. Genetic testing for pathogenic Lamin A variants and subsequent testing against a panel of 77 cardiomyopathy and arrhythmia-related genes were negative.

At 41 months, an echocardiogram demonstrated a mildly dilated LV with mild-moderate systolic impairment (LVEF 45%). Interrogation of his ICD revealed two episodes of NSVT and one fast sustained VT treated by antitachycardia pacing. He was switched to Sotalol for its Class III antiarrhythmic properties and started Ramipril 1.25 mg once daily. Given his electrical and structural phenotype, genetic testing was extended to include FLNC. Subsequently, he tested positive for a likely pathogenic FLNC frameshift variant [c.8107del; p.(Asp2703ThrfsTer69)], thus confirming the diagnosis of ALVC. Predictive testing also uncovered the variant in his three children, all of whom had variable clinical expression of the disease.

**Figure 3:**
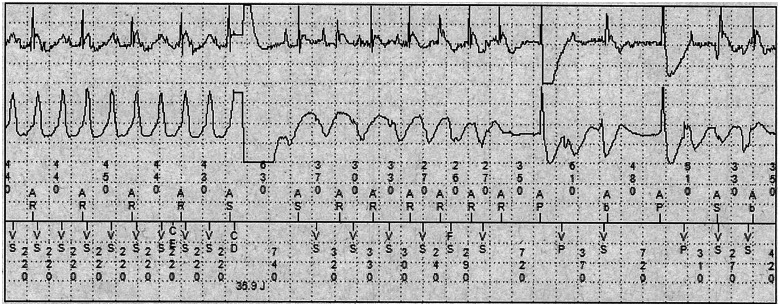
Intracardiac implantable cardioverter-defibrillator electrograms (top channel: atrial near-field; middle channel: ventricular far-field; bottom channel: markers) of an appropriate shock (Charge Delivered (CD) marker) for fast monomorphic ventricular tachycardia (Ventricular sensed (VS) event marker; cycle length 220 ms) occurring within the ventricular fibrillation zone. Recurrence of ventricular tachycardia shortly after shock delivery with intermittent Atrioventricular (AV) sequential pacing, the arrhythmia later spontaneously terminating without the requirement for additional therapy.

At 52 months he was climbing a ski slope and experienced an episode of fast VT requiring an ICD shock (*Figure  3*). Following urgent electrophysiology outpatient review, he was referred for VT ablation utilizing a combined endocardial and epicardial approach. During the procedure, two types of VT arising from the LV were readily induced and radiofrequency ablation was targeted to abnormal local potentials and the region surrounding epicardial and endocardial scar. At 6 months post-ablation, he was asymptomatic and arrhythmia free on Sotalol 80 mg twice daily, and echocardiography showed some improvement in LV systolic function (LVEF 50–54%).

## Discussion

Arrhythmogenic left ventricular cardiomyopathy is an under-characterized phenotype of ACM and few studies have evaluated clinical markers of the disease.^5–7^ The prevalence is also likely underestimated as findings of LV systolic impairment and myocardial fibrosis may overlap clinically with DCM and other ACM phenotypes. This difficulty in characterization was addressed by a statement from the Heart Rhythm Society,^1^ who proposed broadening the definition of ACM to an ‘*arrhythmogenic heart muscle disorder not explained by ischaemic, hypertensive, or valvular heart disease*’*.* The recently introduced *‘Padua criteria’* propose an update from the 2010 Task Force criteria for diagnosing ACM in order to improve differentiation between ACM phenotypes and identification of left-sided genetic causes.^8^^,^^9^ It utilizes a combination of structural, histological, electrocardiographic, and genetic parameters along with the patient’s presentation and family history. For a definitive diagnosis of ALVC, the patient must have a confirmed genetic mutation in one of the genes implicated in disease pathogenesis (*Table 1*).

**Table 1:** Minimum set of genes implicated in the development of arrhythmogenic cardiomyopathy

Gene	Protein type	Mutation type
BLC2-Associated Athanogene 3 (BAG3)	Chaperone	Truncating and missense
Desmin	Intermediate filament	Truncating and missense
Desmocollin-2 (DSC2)	Desmosomal	Truncating and missense
Desmoglein-2 (DSG2)	Desmosomal	Truncating and missense
Desmoplakin (DSP)	Desmosomal	Truncating and missense
Filamin C (FLNC)	Actin crosslink	Truncating and missense
Junction Plakoglobin (JUP)	Desmosomal	Missense
LIM Domain Binding 3 (LBD3)	Z-band	Missense
Lamin A/C (LMNA)	Nuclear envelope	Truncating and missense
NK2 Homeobox 5 (NKX2-5)	Homeobox	Truncating and missense
Plakophilin-2 (PKP2)	Desmosomal	Truncating
Phospholamban (PLN)	Calcium handing	Missense, nonsense, and deletion
RNA-Binding Motif Protein 20 (RBM20)	Splice factor	Missense
Sodium Voltage-Gated Channel Alpha Subunit 5 (SCN5A)	Sodium channel	Mostly missense
Transmembrane Protein 43 (TMEM43)	Nuclear envelope	Missense

Adapted from HRS guidelines.^1^

The presenting feature in this case was an initially unexplained cardiac arrest secondary to ventricular arrhythmia. In conjunction with the scarring pattern on CMRI, a diagnosis of ALVC was suspected but could not be confirmed without genetic evidence. Although cardiovascular magnetic resonance has a fundamental role in delineating phenotype, additional investigative modalities such as molecular genetic studies, family screening, and rarely endomyocardial biopsy, may improve diagnostic certainty. Other suggestive features of ALVC include the presence of inferolateral T-wave inversion or apparent DCM with an arrhythmic burden incongruous with myocardial function.^7^

The elusive genetic basis of this case supports evolving evidence that the genetic architecture underlying ACM are more complex than previously thought. Consequently, the use of existing and potentially ‘outdated’ gene panels may omit newly identified genes that are implicated in disease pathogenesis, thereby precluding a formal diagnosis. Therefore, it is prudent for clinicians to have a low threshold for broadened genetic testing in patients with a suggestive phenotype, as seen here where the decision to extend gene testing yielded the diagnosis of FLNC-induced ALVC. Notably, our patient’s variant had been reported in two other patients with comparable phenotypes.^10^^,^^11^

Whilst rare, ACM-associated FLNC presents with a distinctive phenotype of abnormalities including LV dilation and myocardial fibrosis, systolic dysfunction, inferolateral negative T waves, low QRS voltages, and ventricular arrhythmias,^1^^,^^3^^,^^10^ all of which were present in this case. Truncating variants in FLNC have been shown to exhibit a dominant inheritance pattern and high penetrance amongst genotype-positive individuals (>97% in carriers older than 40 years of age).^10^ As such, extending genetic testing to family members is essential to allow for early intervention. Moreover, genetic testing can occasionally enhance risk stratification and management. In lower risk FLNC–ACM patients without a history of sustained arrhythmia, international consensus currently supports ICD implantation in those exhibiting moderate LV systolic impairment (LVEF < 45%).^1^

Overall, the aim of medical therapy in ACM is to treat arrhythmia, optimize ventricular function, and manage symptoms.^1^ Exercise is also known to accelerate disease progression and competitive sport is associated with an increased risk of ventricular arrhythmia and SCD.^12^ Several observational studies also suggest a dose-dependent relationship between participation in endurance exercise and risk of developing ACM in genotype-positive family members.^1^^,^^13^ Therefore, a comprehensive therapeutic approach includes restriction from endurance and high intensity sports, medical therapy (including antiarrhythmic agents and prognostic medications in patients with LV systolic impairment) and ICD implantation, where indicated. Ventricular tachycardia ablation may also be utilized to good effect, as seen here where our patient experienced multiple ICD-treated arrhythmias despite optimized medical therapy.

## Lead author biography

**Figure:**
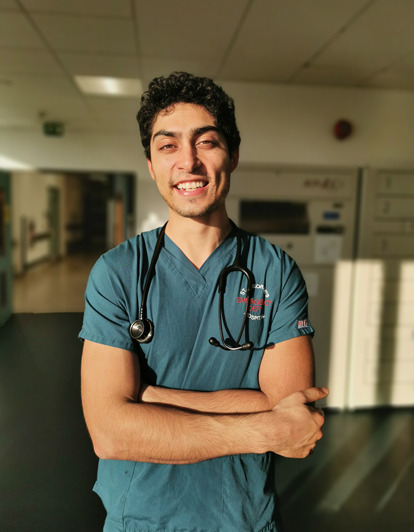


Navneet Kandhari graduated from Newcastle University with an MBBS and MRes degree in 2019. He obtained a PgDip in Medical Education in 2021. He now works as a junior doctor in the UK. His current interests include cardiology, academic research, and medical education. 

##  Supplementary material

Supplementary material is available at *European Heart Journal - Case Reports* online.

## Supplementary Material

ytab422_Supplementary_Data
