# Supplementary material for: Cardiac arrest as first presentation of arrhythmogenic left ventricular cardiomyopathy due to Filamin C mutation: a case report
Source: Eur Heart J Case Rep. 2021 Nov 22;5(11):ytab422. doi: 10.1093/ehjcr/ytab422 (PMC8728717; doi:10.1093/ehjcr/ytab422)
Supplement: ytab422_Supplementary_Data [file ytab422_Supplementary_Data.zip › ACM case report slide set V3.pptx]

## Slide 1
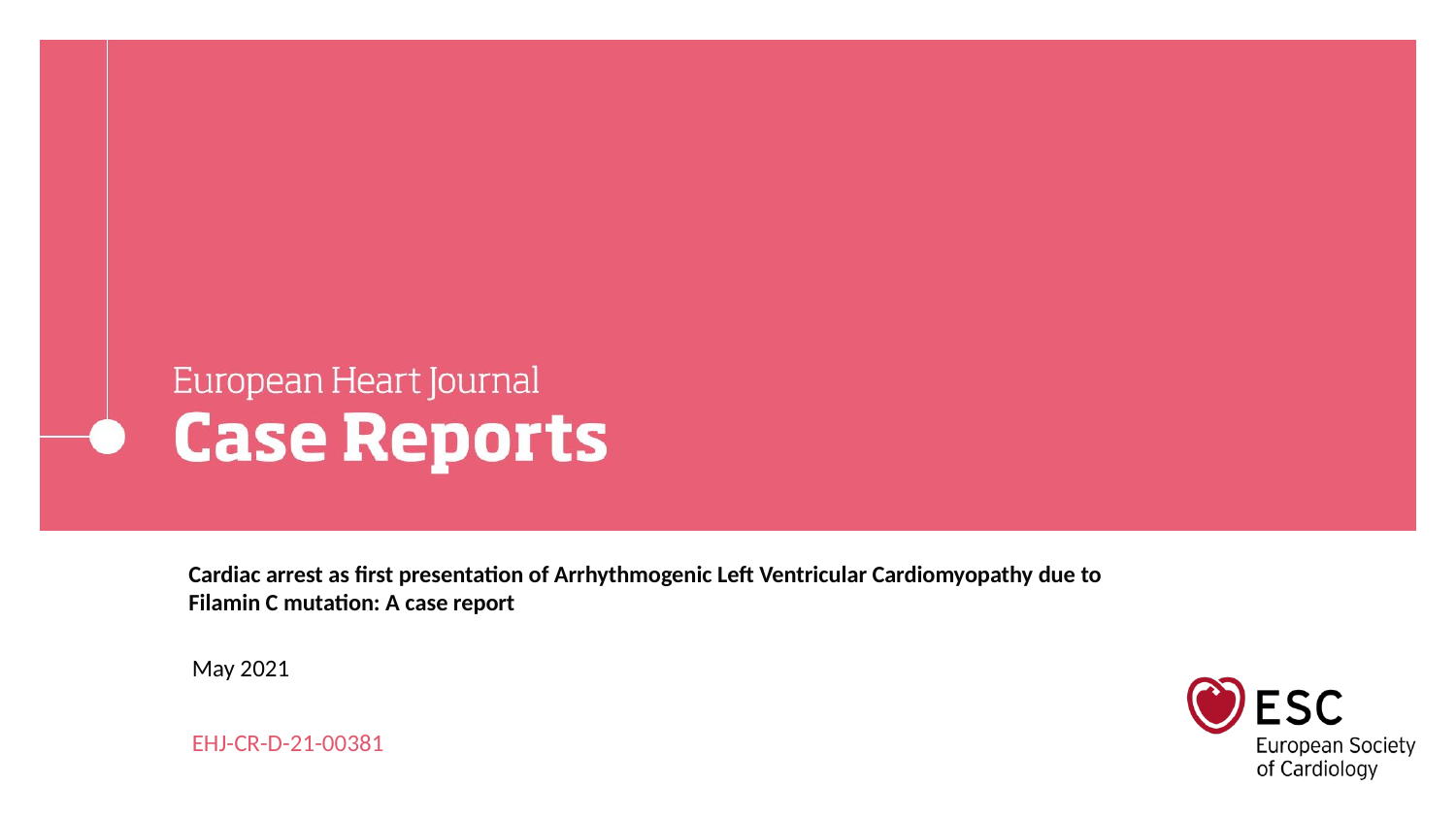

# Cardiac arrest as first presentation of Arrhythmogenic Left Ventricular Cardiomyopathy due to Filamin C mutation: A case report
May 2021
EHJ-CR-D-21-00381

## Slide 2
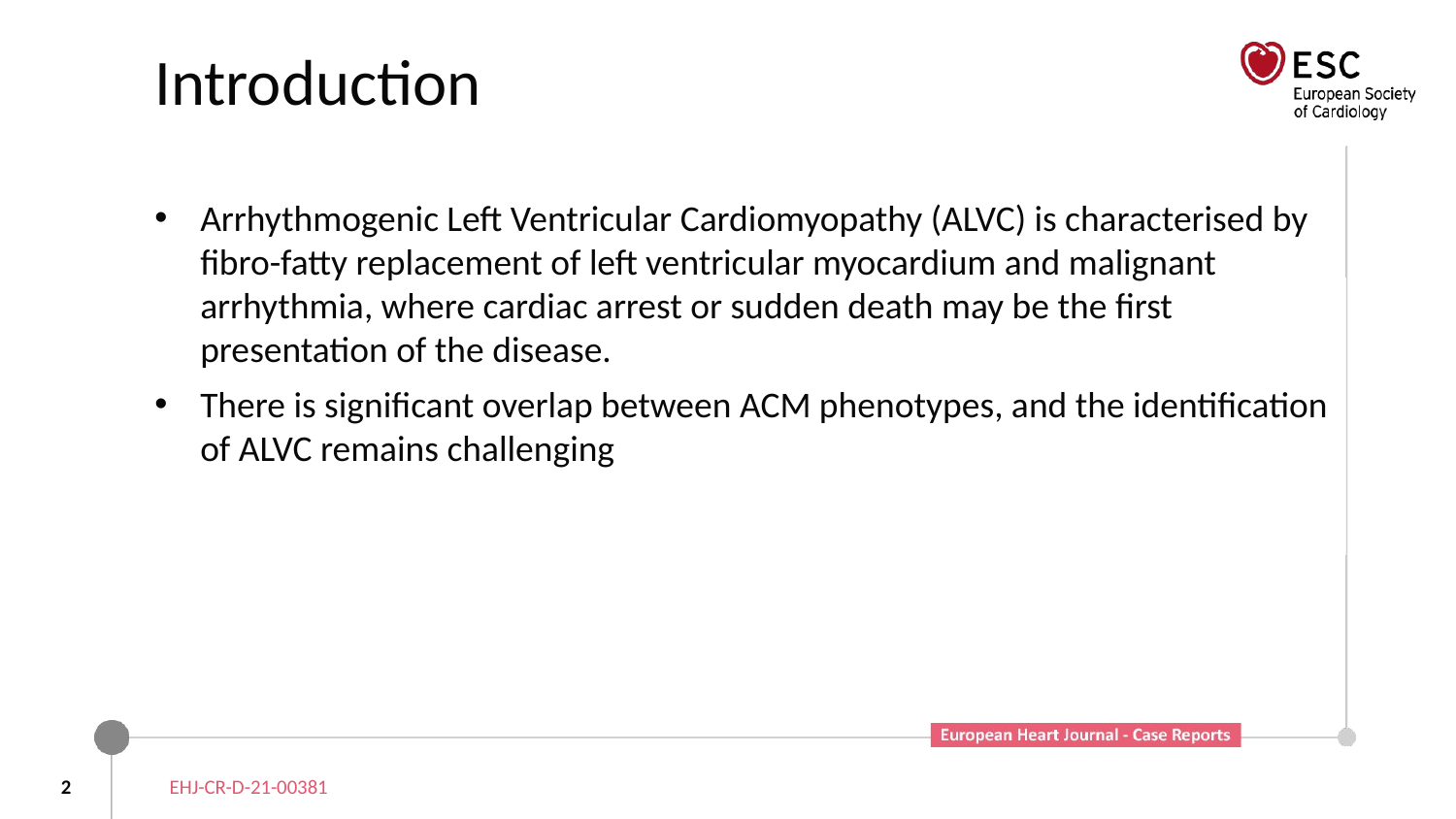

# Introduction
Arrhythmogenic Left Ventricular Cardiomyopathy (ALVC) is characterised by fibro-fatty replacement of left ventricular myocardium and malignant arrhythmia, where cardiac arrest or sudden death may be the first presentation of the disease.
There is significant overlap between ACM phenotypes, and the identification of ALVC remains challenging
2
EHJ-CR-D-21-00381

## Slide 3
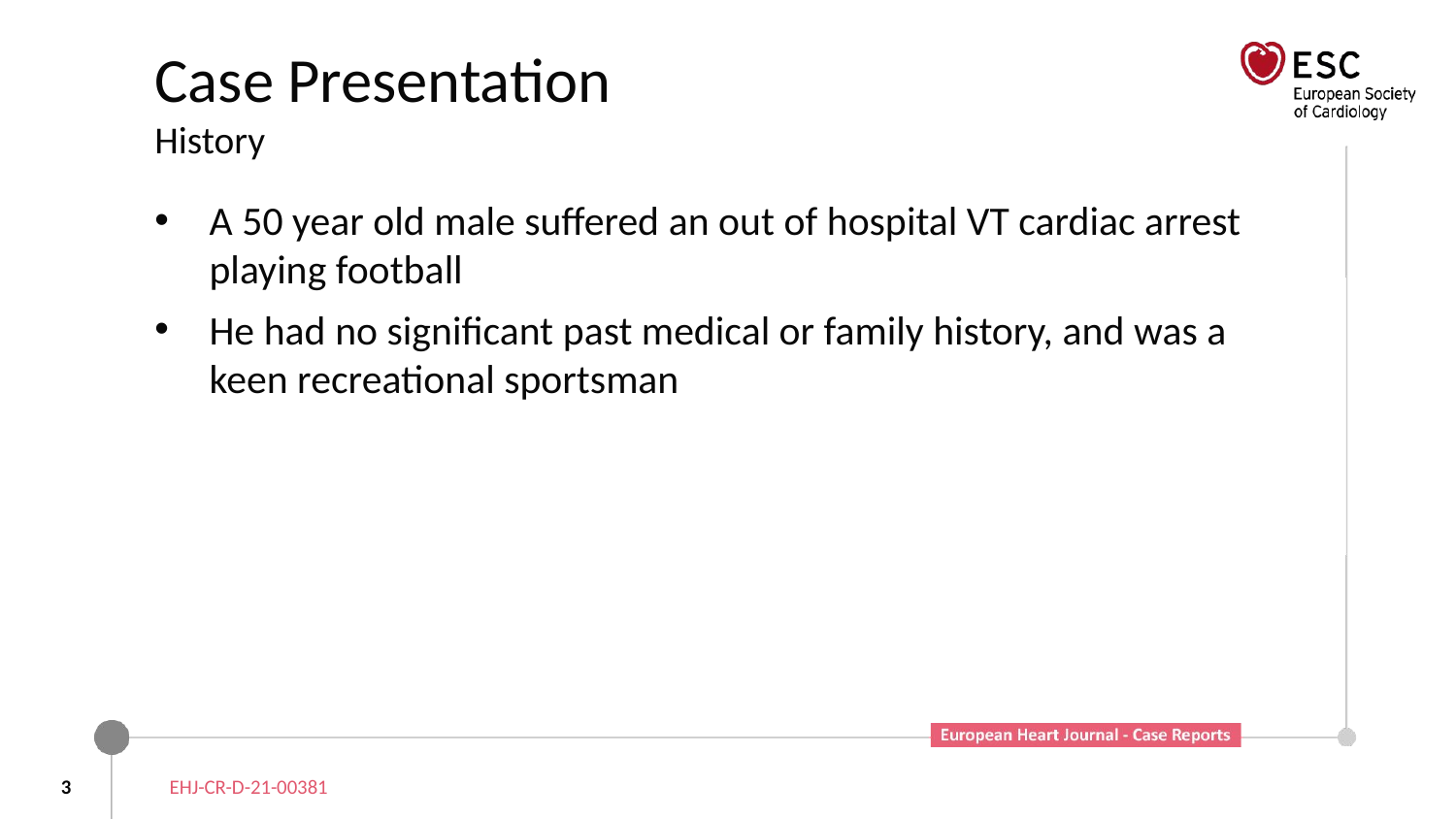

# Case Presentation History
A 50 year old male suffered an out of hospital VT cardiac arrest playing football
He had no significant past medical or family history, and was a keen recreational sportsman
3
EHJ-CR-D-21-00381

## Slide 4
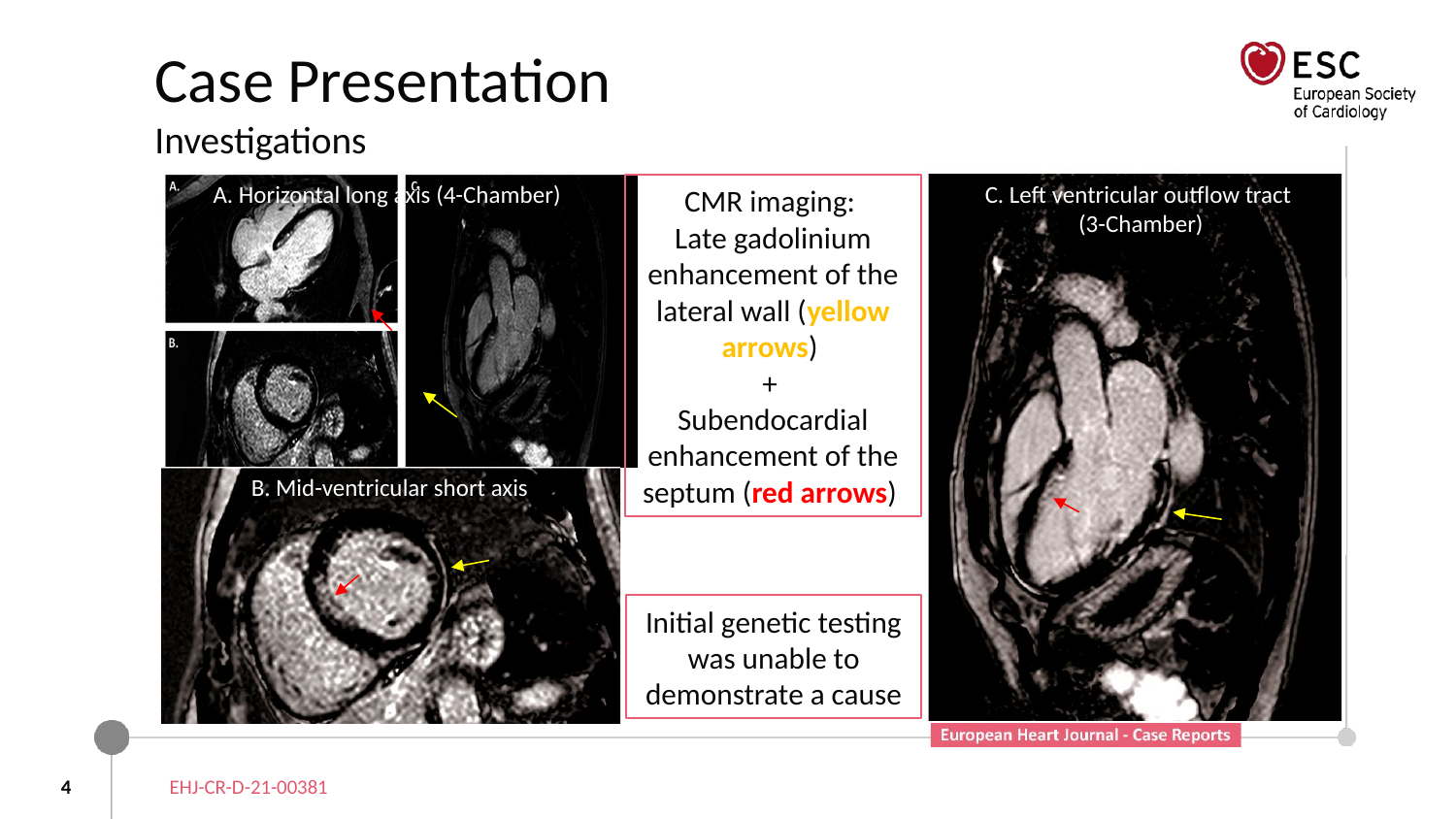

# Case Presentation Investigations
A. Horizontal long axis (4-Chamber)
C. Left ventricular outflow tract
(3-Chamber)
CMR imaging:
Late gadolinium enhancement of the lateral wall (yellow arrows)
+
Subendocardial enhancement of the septum (red arrows)
B. Mid-ventricular short axis
Initial genetic testing was unable to demonstrate a cause
4
EHJ-CR-D-21-00381

## Slide 5
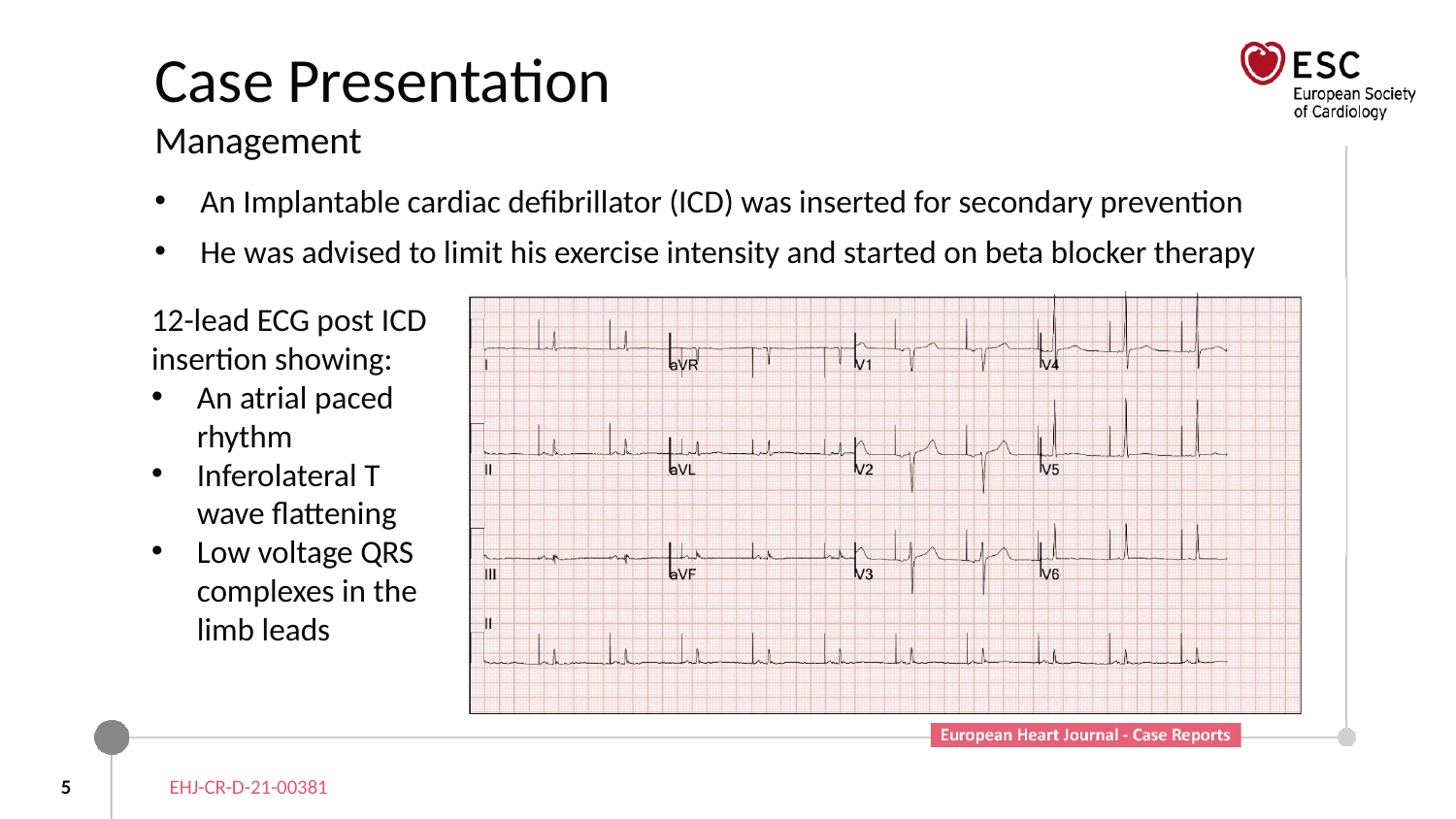

# Case Presentation Management
An Implantable cardiac defibrillator (ICD) was inserted for secondary prevention
He was advised to limit his exercise intensity and started on beta blocker therapy
12-lead ECG post ICD insertion showing:
An atrial paced rhythm
Inferolateral T wave flattening
Low voltage QRS complexes in the limb leads
5
EHJ-CR-D-21-00381

## Slide 6
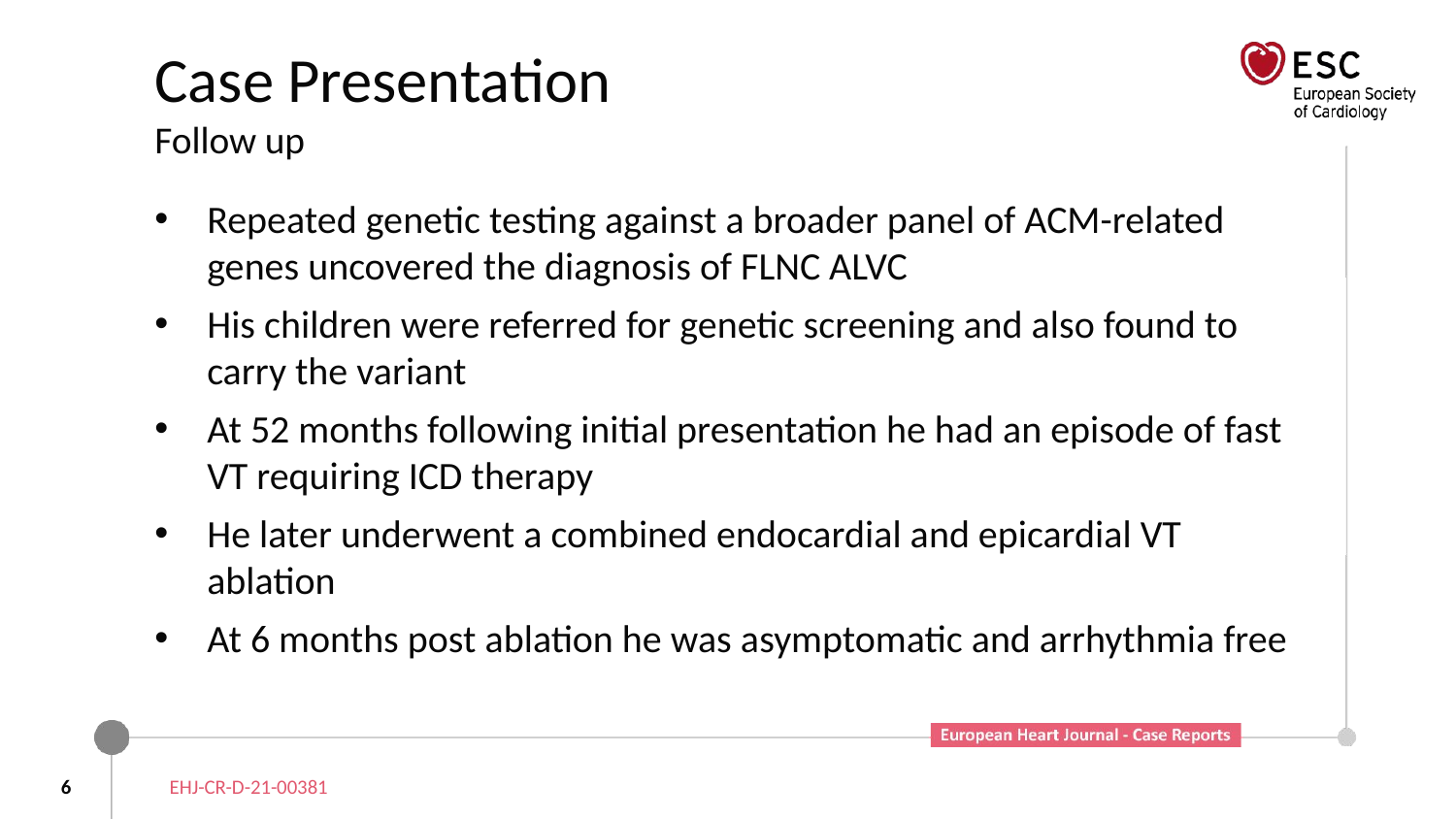

# Case Presentation Follow up
Repeated genetic testing against a broader panel of ACM-related genes uncovered the diagnosis of FLNC ALVC
His children were referred for genetic screening and also found to carry the variant
At 52 months following initial presentation he had an episode of fast VT requiring ICD therapy
He later underwent a combined endocardial and epicardial VT ablation
At 6 months post ablation he was asymptomatic and arrhythmia free
6
EHJ-CR-D-21-00381

## Slide 7
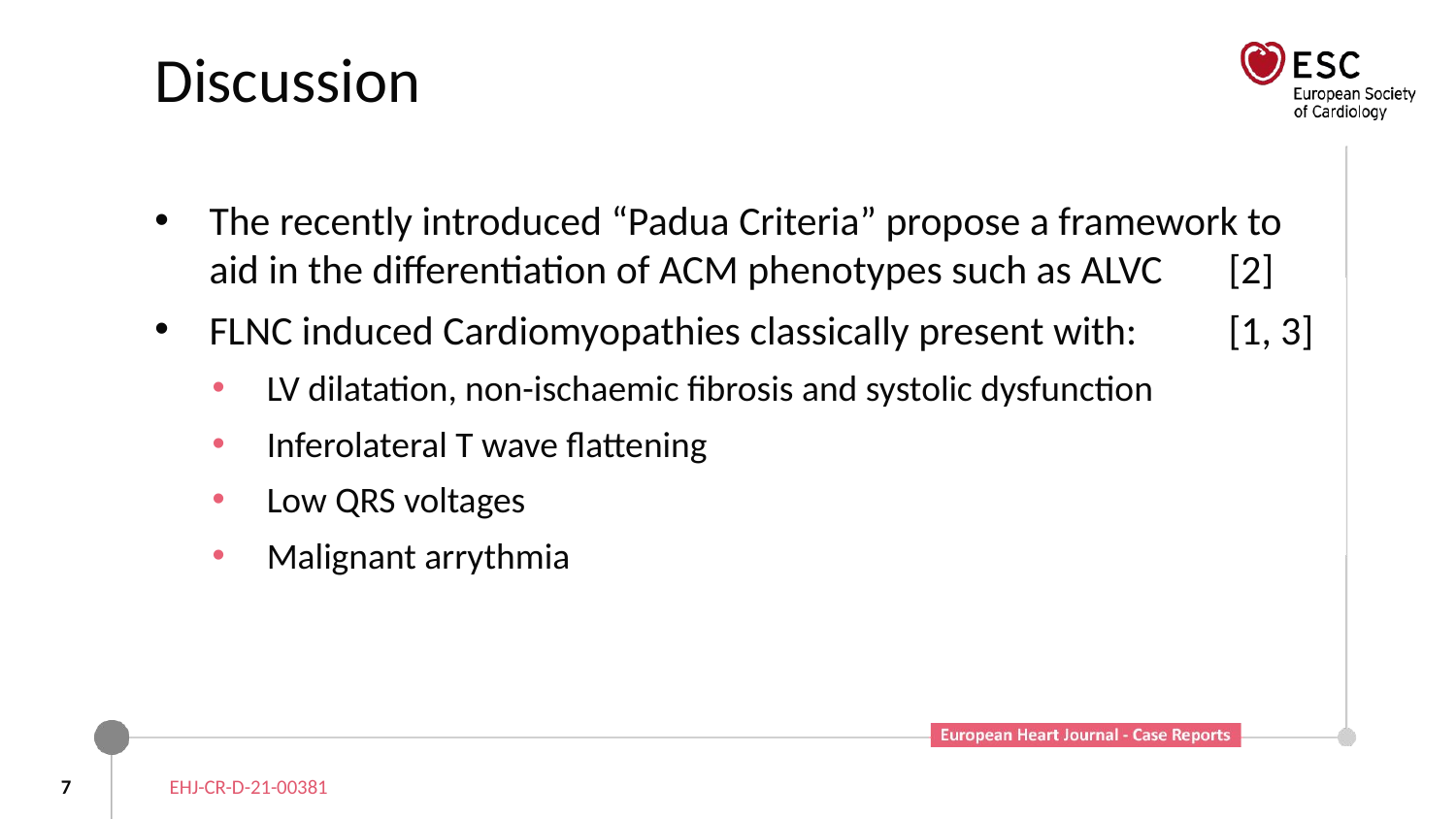

# Discussion
The recently introduced “Padua Criteria” propose a framework to aid in the differentiation of ACM phenotypes such as ALVC 	[2]
FLNC induced Cardiomyopathies classically present with: 	[1, 3]
LV dilatation, non-ischaemic fibrosis and systolic dysfunction
Inferolateral T wave flattening
Low QRS voltages
Malignant arrythmia
7
EHJ-CR-D-21-00381

## Slide 8
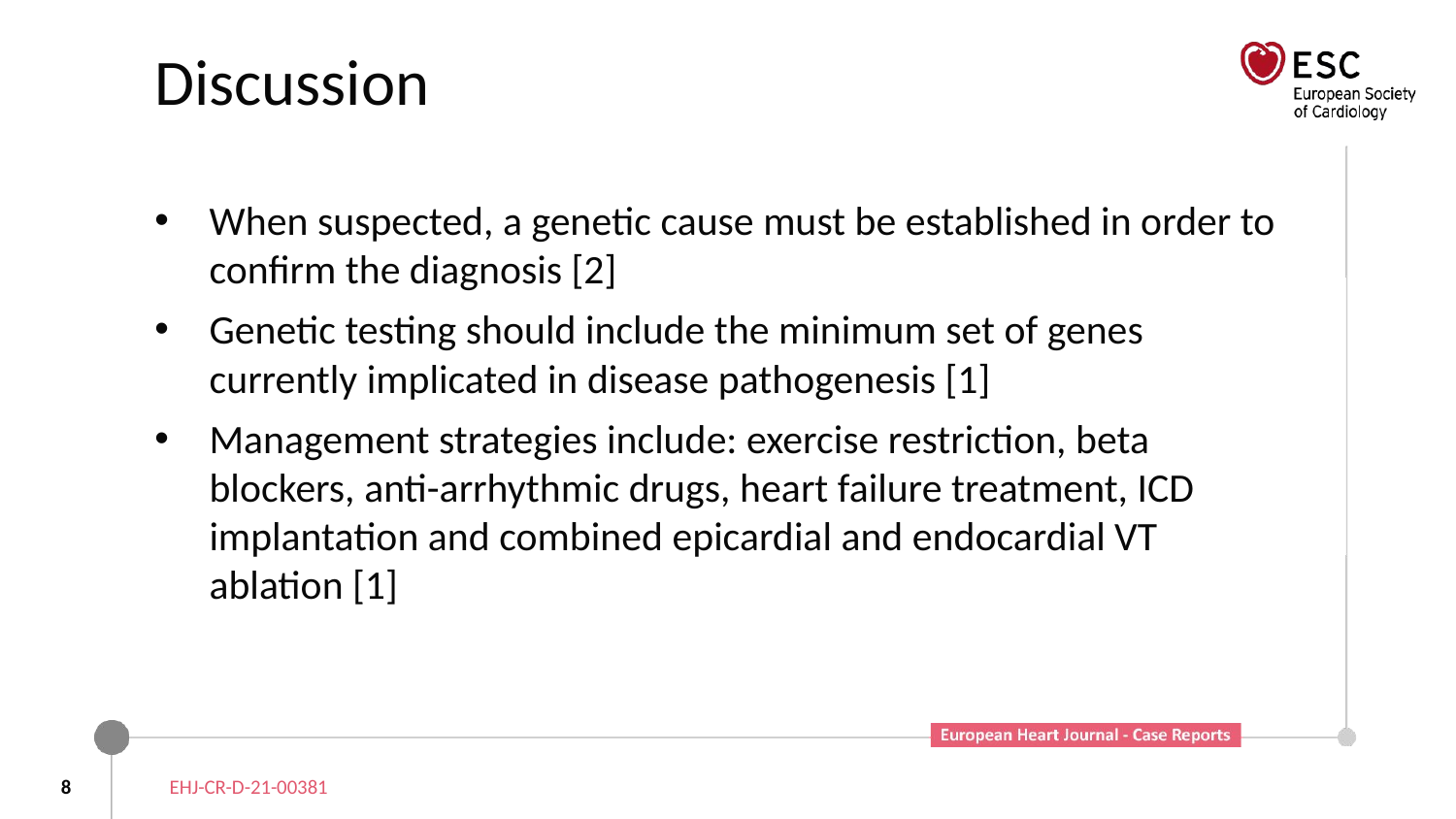

# Discussion
When suspected, a genetic cause must be established in order to confirm the diagnosis [2]
Genetic testing should include the minimum set of genes currently implicated in disease pathogenesis [1]
Management strategies include: exercise restriction, beta blockers, anti-arrhythmic drugs, heart failure treatment, ICD implantation and combined epicardial and endocardial VT ablation [1]
8
EHJ-CR-D-21-00381

## Slide 9
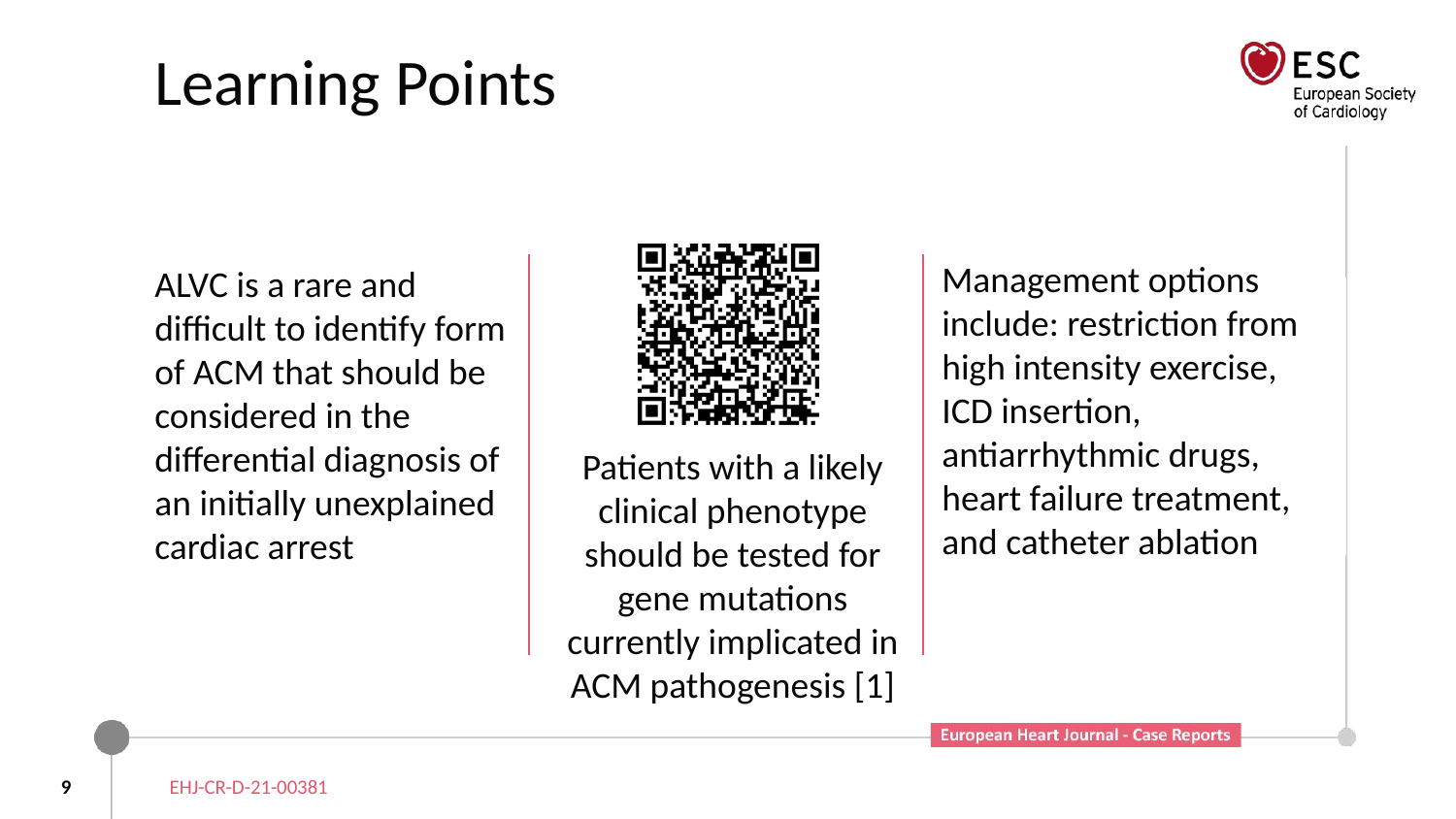

# Learning Points
Management options include: restriction from high intensity exercise, ICD insertion, antiarrhythmic drugs, heart failure treatment, and catheter ablation
ALVC is a rare and difficult to identify form of ACM that should be considered in the differential diagnosis of an initially unexplained cardiac arrest
Patients with a likely clinical phenotype should be tested for gene mutations currently implicated in ACM pathogenesis [1]
9
EHJ-CR-D-21-00381

## Slide 10
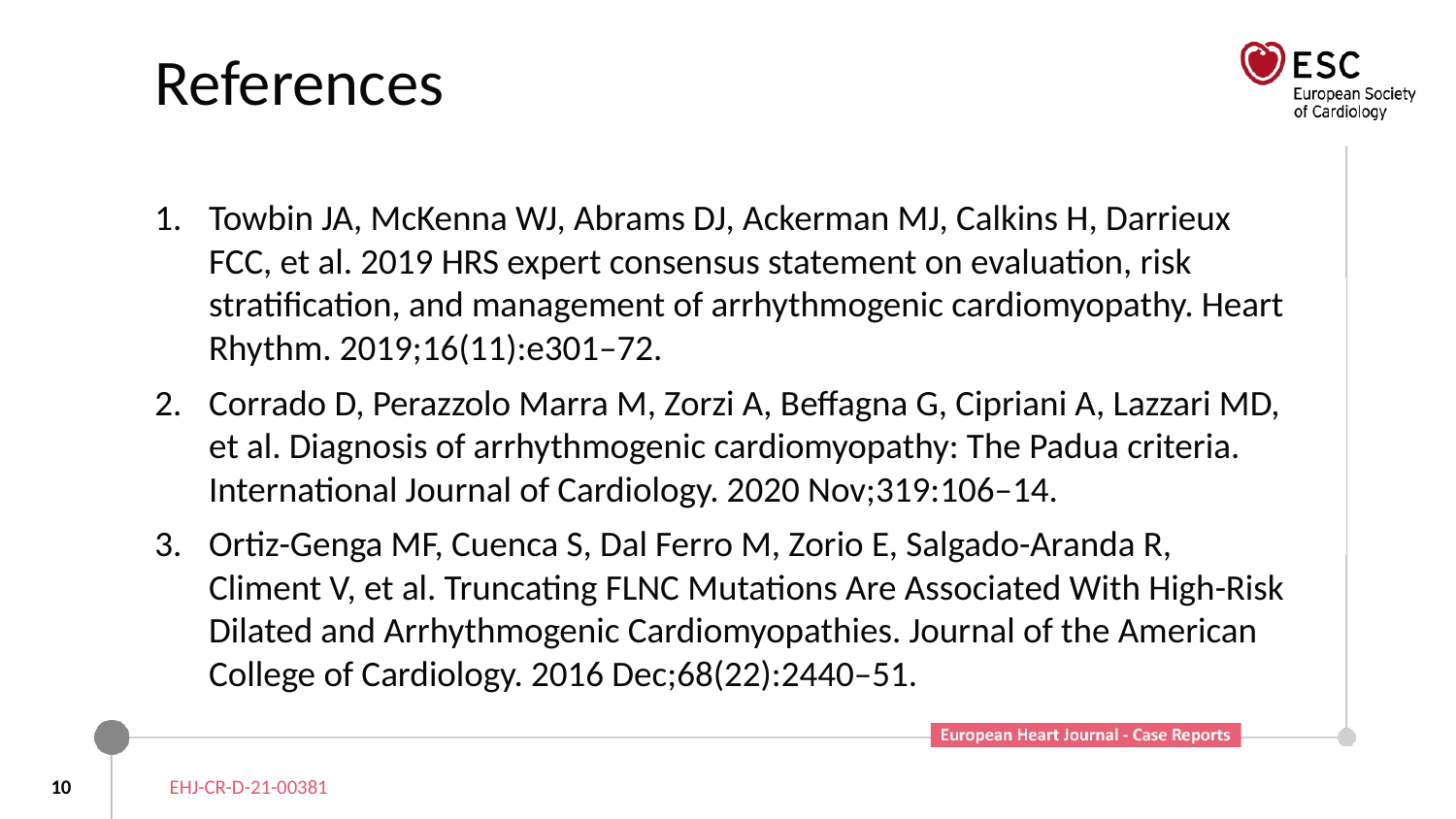

# References
Towbin JA, McKenna WJ, Abrams DJ, Ackerman MJ, Calkins H, Darrieux FCC, et al. 2019 HRS expert consensus statement on evaluation, risk stratification, and management of arrhythmogenic cardiomyopathy. Heart Rhythm. 2019;16(11):e301–72.
Corrado D, Perazzolo Marra M, Zorzi A, Beffagna G, Cipriani A, Lazzari MD, et al. Diagnosis of arrhythmogenic cardiomyopathy: The Padua criteria. International Journal of Cardiology. 2020 Nov;319:106–14.
Ortiz-Genga MF, Cuenca S, Dal Ferro M, Zorio E, Salgado-Aranda R, Climent V, et al. Truncating FLNC Mutations Are Associated With High-Risk Dilated and Arrhythmogenic Cardiomyopathies. Journal of the American College of Cardiology. 2016 Dec;68(22):2440–51.
10
EHJ-CR-D-21-00381
